# The Role of Biodegradable Poly-(L-lactide)-Based Polymers in Blood Cell Activation and Platelet-Monocyte Interaction

**DOI:** 10.3390/ijms22126340

**Published:** 2021-06-13

**Authors:** Anne Strohbach, Friedemann Maess, Katharina Wulf, Svea Petersen, Niels Grabow, Klaus-Peter Schmitz, Stephan B. Felix, Raila Busch

**Affiliations:** 1 Department of Internal Medicine B Cardiology, University Medicine Greifswald, Ferdinand-Sauerbruch-Str., 17475 Greifswald, Germany; strohbac@uni-greifswald.de (F.M.); Stephan.Felix@med.uni-greifswald.de (S.B.F.); buschr@uni-greifswald.de (R.B.); 2 DZHK (German Centre for Cardiovascular Research), Partner Site Greifswald, Fleischmannstr. 42-44, 17489 Greifswald, Germany; 3 Institute for Biomedical Engineering, Rostock University Medical Center, Friedrich-Barnewitz-Str. 4, 18119 Rostock, Germany; katharina.wulf@uni-rostock.de (K.W.); s.petersen@hs-osnabrueck.de (S.P.); niels.grabow@uni-rostock.de (N.G.); klaus-peter.schmitz@uni-rostock.de (K.-P.S.); 4 Faculty of Engineering and Computer Science, University of Applied Sciences, Albrechtstr. 30, 49076 Osnabrück, Germany

**Keywords:** poly-(L-lactide), platelet activation, leukocyte activation, platelet–monocyte aggregates, shear stress

## Abstract

The main purpose of new stent technologies is to overcome unfavorable material-related incompatibilities by producing bio- and hemo-compatible polymers with anti-inflammatory and anti-thrombogenic properties. In this context, wettability is an important surface property, which has a major impact on the biological response of blood cells. However, the influence of local hemodynamic changes also influences blood cell activation. Therefore, we investigated biodegradable polymers with different wettability to identify possible aspects for a better prediction of blood compatibility. We applied shear rates of 100 s^−1^ and 1500 s^−1^ and assessed platelet and monocyte activation as well as the formation of CD62P+ monocyte-bound platelets via flow cytometry. Aggregation of circulating platelets induced by collagen was assessed by light transmission aggregometry. Via live cell imaging, leukocytes were tracked on biomaterial surfaces to assess their average velocity. Monocyte adhesion on biomaterials was determined by fluorescence microscopy. In response to low shear rates of 100 s^−1^, activation of circulating platelets and monocytes as well as the formation of CD62P+ monocyte-bound platelets corresponded to the wettability of the underlying material with the most favorable conditions on more hydrophilic surfaces. Under high shear rates, however, blood compatibility cannot only be predicted by the concept of wettability. We assume that the mechanisms of blood cell-polymer interactions do not allow for a rule-of-thumb prediction of the blood compatibility of a material, which makes extensive in vitro testing mandatory.

## 1. Introduction

Using bioresorbable polymers for new stent technologies has been a revolutionary advance in the therapy of cardiovascular diseases (CVD). Nevertheless, in-stent restenosis (ISR) and stent thrombosis (ST) still represent the main adverse reactions to coronary stents. A major step forward was achieved with the advent of current second-generation DES (2G-DES). Amongst other strategies, these incorporate more biocompatible or biodegradable polymers with an associated decrease in ISR, ST, duration of dual anti-platelet therapy, and bleeding (reviewed in [1,2]). The further development of 2G-DES, however, focuses on biodegradable polymer carriers or fully biodegradable vascular scaffolds (BVS), which degrade during or after drug release. The most frequently used polymers for DES application are polylactides (PLA), due to their unique ability to be completely resorbed in vivo in a material-specific time frame ranging from months to a few years [3]. Despite its high biocompatibility, PLA has been discussed in the context of hypersensitivity reactions, stent thrombosis and in-stent-restenosis [4]. One strategy to overcome unfavorable material-related incompatibility is the formation of a biological inert surface [1], which can be achieved, for example, by copolymerization/blending with other polymers [5].

The main purpose of new stent technologies is to produce bio- and hemo-compatible polymers to reduce immunologic reactions and facilitate reendothelialization in the stented region [6]. Regarding blood compatibility, it is particularly important that the surface shows anti-thrombogenic properties to prevent thrombosis and the possibility of bleeding after implantation [7]. In this context, wettability is an important surface property, which has a major impact on the biological response and is often associated with the adsorption of proteins [8,9,10,11,12]. Thereby, wettability not only affects the amount of adsorbed protein but also the conformation, which possibly determines blood cell activation and clot formation [1,2]. Artificial surfaces that come into contact with blood induce an immediate activation of the coagulation cascade, leading to a thrombotic and/or inflammatory response that can eventually cause damage to the implant or the patient, or to both [13,14]. Hence, it is even more important to understand the underlying mechanisms of material-induced coagulation and thrombosis [15]. Especially after deposition of an implant, which leads to denudation of the vessel wall, leukocyte activation can occur through complement components [16]. Activated leukocytes further contribute to thrombosis through pro-coagulant properties such as the upregulation of the α–Μ integrin CD11b or the formation of leukocyte-platelet aggregates [15]. In this context, the effect of shear stress on blood cell activation is well recognized. While platelet activation proportionally correlates with the magnitude of shear rates [17], physiological shear rates ranging from 1000 to 10,000 s^−1^ cannot activate leukocytes [18]. However, the presence of a biomaterial might well be a potential stimulus for blood cells triggering cell activation and adhesion even under physiological shear rates. Despite numerous studies conducted to evaluate blood compatibility, there are no standardized methods for testing the hemocompatibility of available biomaterials [19]. 

In this context, we applied shear rates of 100 s^−1^ and 1500 s^−1^ to study the effect of different flow conditions on blood cell activation in the presence of poly-(L-lactide) (PLLA)–based biomaterials: PLLA, poly(4-hydroxybutyrate) P(4HB), PLLA/P(4HB), poly-ϵ-caprolactone, and PLLA/PCL. Platelet adhesion and aggregation as well as leukocyte motility and monocyte adhesion, were investigated. Additionally, a physiological suspension of human platelets and leukocytes was perfused over the polymeric surfaces and flow cytometry was used to characterize blood cell activation and leukocyte–platelet aggregates. 

## 2. Results

### 2.1. Polymer Wettability

Contact angle (Θ) measurements of the polymer films revealed minor differences in wettability as shown in Table 1. Thereby, lower contact angles translate into higher wettability and less hydrophobicity of the material. However, the combination of PLLA with P(4HB) significantly reduced wettability compared to PLLA alone (PLLA/P(4HB) vs. PLLA: 76.7 ± 2.8° vs. 82.2 ± 1.8, *p* = 0.001).

### 2.2. Platelet–Polymer Interaction

Since some polymers seem likely to promote the formation of platelet aggregates, we investigated the influence of the polymers on platelet aggregation and activation by analyzing P-selectin expression under flow conditions with shear rates of 100 s^−1^, simulating flow rates of recirculation zones and between stent struts, and 1500 s^−1^, simulating arterial flow conditions over stent struts (in the following referred to as “low flow” and “high flow”, respectively). 

**Figure 1:**
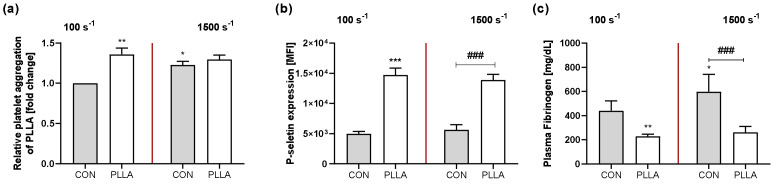
PLLA influences platelet aggregation and activation. Human platelets were circulated through a parallel plate flow chamber system, containing either no polymer or the indicated materials, for 5 min with shear rates of 100 s^−1^ and 1500 s^−1^: (**a**) After flow exposure, collagen-induced platelet aggregation was measured. (**b**) Subsequent FACS analysis was performed immediately after flow exposure. Therefore, PRP was collected and platelets were stained for P-selectin. (**c**) PRP was collected for ELISA measurement of soluble fibrinogen. Bars show mean ± SD of 5 independent experiments compared to a polymer-free control. Significances to the corresponding control are given with * *p* < 0.05, ** *p* < 0.01 and *** *p* < 0.001. Significances between groups are given as ### *p* < 0.0001.

First, we characterized platelets circulating in a polymer-free flow chamber system (Figure S1) demonstrating that platelet aggregation and activation are not significantly altered when exposed to low shear rates (no shear vs. 100 s^−1^: aggregation: 68.0 ± 8.7% vs. 71.5 ± 11.0%, *p* = 0.775; MFI of P-selectin: 4.3 ± 0.4 × 10^3^ vs. 5.0 ± 0.4 × 10^3^, *p* = 0.268; plasma Fibrinogen (Fg): 305.4 ± 82.9 mg/dL vs. 440.5 ± 81.3 mg/dL, *p* = 0.120). Under high flow conditions, however, platelet aggregation and activation increase (no shear vs. 1500 s^−1^: aggregation: 68.0 ± 8.7% vs. 85.7 ± 5.8%, *p* = 0.013; MFI of P-selectin: 4.3 ± 0.4 × 10^3^ vs. 5.6 ± 0.8 × 10^3^, *p* = 0.021; plasma Fg: 305.4 ± 82.9 mg/dL vs. 598.0 ± 144.4 mg/dL, *p* = 0.001). Compared to the polymer-free control, the presence of the biomaterial PLLA influences platelet aggregation and activation (Figure 1). Under low flow, PLLA promotes platelet aggregation by 36% *p* = 0.002), promotes surface P-selectin expression (5.0 ± 0.4 × 10^3^ vs. 14.7 ± 1.1 × 10^3^, *p* < 0.0001), and reduces plasma Fg concentration after perfusion (440.5 ± 81.3 mg/dL vs. 228.1 ± 19.3 mg/dL, *p* = 0.007). Under high flow conditions, PLLA has no additional effect on platelet aggregation (CON vs. PLLA: 1.23 ± 0.05 vs. 1.30 ± 0.06; *p* = 0.590), but induces a significantly increased expression of surface P-selectin on platelets (5.6 ± 0.8 × 10^3^ vs. 13.9 ± 0.9 × 10^3^, *p* < 0.0001), while significantly reducing plasma Fg (598.0 ± 144.4 mg/dL vs. 261.6 ± 49.5 mg/dL, *p* < 0.0001).

**Figure 2:**
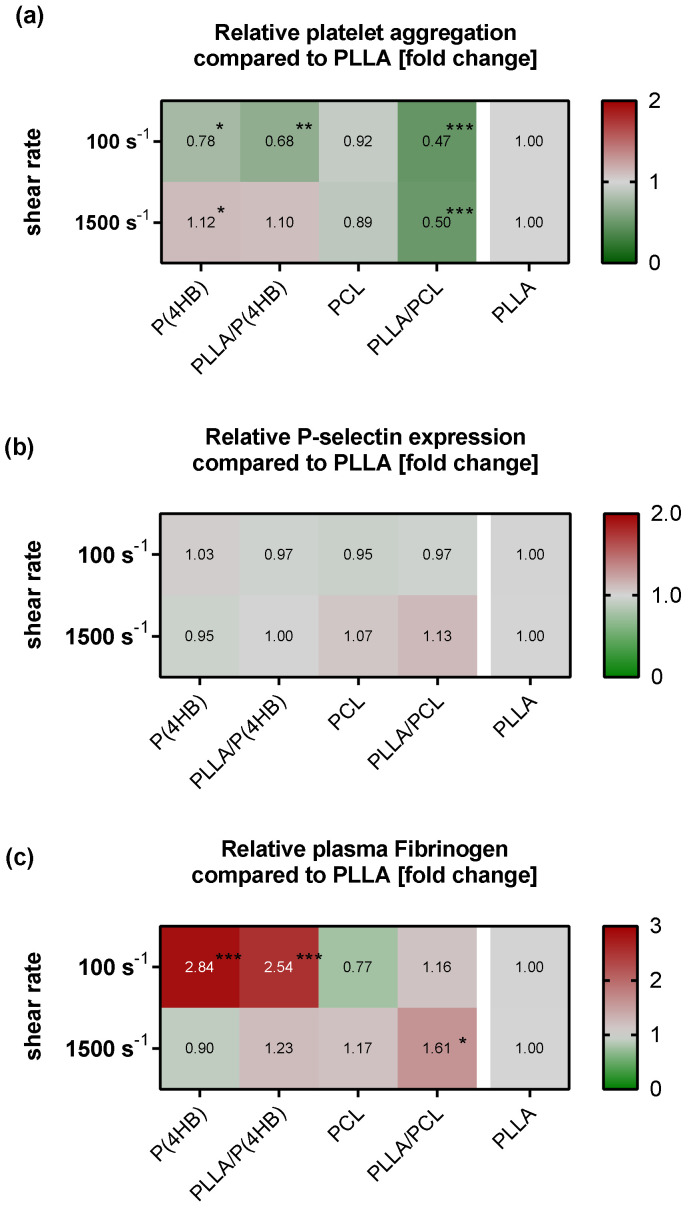
Material characteristics alter platelet response to polymers. Human platelets were circulated through a parallel plate flow chamber system, containing either no polymer or the indicated materials, for 5 min with shear rates of 100 s^−1^ and 1500 s^−1^: (**a**) After flow exposure, collagen-induced platelet aggregation was measured. (**b**) Subsequent FACS analysis was performed immediately after flow exposure. Therefore, PRP was collected and platelets were stained for P-selectin. (**c**) PRP was collected for ELISA measurement of soluble fibrinogen. To visualize the effect of the different polymers, heat maps show the relative mean of at least five independent experiments compared to PLLA. Significances with regard to PLLA are indicated, with * *p* < 0.05, ** *p* < 0.01 and *** *p* < 0.001.

Further, platelet aggregation and activation are altered by the polymers under both shear conditions (Figure 2). Statistical analysis of our results with a two way-ANOVA model revealed that the aggregation of platelets is not only influenced by the applied flow conditions (*p* < 0.0001) but also by the polymers (*p* < 0.0001) with a significant interaction of both parameters (*p* < 0.0001). Compared to PLLA, shear-induced platelet aggregation increases with decreasing wettability under low flow conditions (P(4HB): 0.78 ± 0.03, *p* = 0.029; PLLA/P(4HB): 0.68 ± 0.06, *p* = 0.001; PCL: 0.92 ± 0.04, *p* = 0.594) except for PLLA/PCL, where shear-induced platelet aggregation is most reduced (0.47 ± 0.12, *p* < 0.0001; Figure 2a). Regarding high flow, the effect on shear-induced platelet aggregation tends to be opposite (P(4HB): 1.18 ± 0.03, *p* = 0.048; PLLA/P(4HB): 1.10 ± 0.05, *p* = 0.136; PCL: 0.89 ± 0.1, *p* = 0.065) again with an exception for PLLA/PCL, which reduces platelet aggregation significantly (0.50 ± 0.13; *p* < 0.0001; Figure 2a). While aggregation is influenced by both, shear forces as well as the material, platelet P-selectin expression is not influenced compared to PLLA, keeping in mind, that platelets in the presence of all polymers are strongly activated compared to the polymer-free control (Figure 2b). Plasma Fg is again significantly influenced by the applied shear forces (*p* < 0.0001) and the underlying polymer (*p* < 0.0001) with a significant interaction for both parameters (*p* < 0.0001). Compared to PLLA, Fg level in plasma decreases with decreasing wettability under low flow conditions (P(4HB): 2.84 ± 0.2, *p* < 0.0001; PLLA/P(4HB): 2.54 ± 0.7, *p* < 0.0001; PCL: 0.77 ± 0.1, *p* = 0.559; PLLA/PCL: 1.16 ± 0.3, *p* = 0.858; Figure 2c). Under high flow, Fg concentration in plasma is similar to PLLA on all polymers except PLLA/PCL (P(4HB): 0.90 ± 0.1, *p* = 0.981; PLLA/P(4HB): 1.23 ± 0.2, *p*= 0.612; PCL: 1.17 ± 0.3, *p* = 0.824; PLLA/PCL: 1.61 ± 0.4, *p* = 0.026; Figure 2c). Absolute Fg levels measured in plasma after perfusion in the presence of the polymers are given in Table S1.

### 2.3. Leukocyte–Polymer Interaction

**Figure 3:**
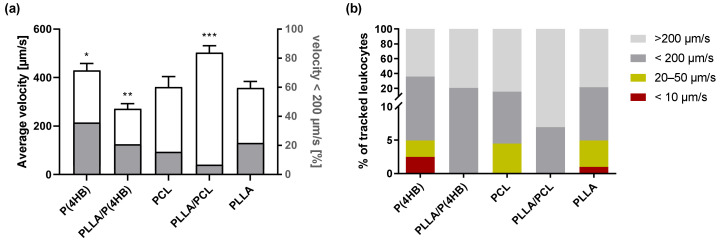
Material-dependent leukocyte movement. Human peripheral blood mononuclear cells (PBMCs) were isolated from an in-line filter system for leukocyte filtration, stained with Calcein Red-Orange, AM and exposed to laminar flow with a shear rate of 50 s^−1^ for 5 min. (**a**) Average velocity of leukocytes was tracked using NIS-Elements AR Imaging Software. Bars show mean average velocity ± SD of 5 independent experiments compared to the PLLA surface with * *p* < 0.05, ** *p* < 0.01 and *** *p* < 0.001. (**b**) All tracked leukocytes were grouped regarding their velocity to vizualize velocity distribution on the different polymers.

The interactions with stent materials and vascular injury caused by stent placement have been shown to induce leukocyte activation [20]. In fact, ISR after stent implantation constitutes the earliest manifestation of an inflammatory reaction, where tethering and rolling of circulating leukocytes are the initial adhesive interactions with the implanted material. In this context, we determined the velocity of leukocytes on polymer surfaces under flow conditions (Figure 3). Our data demonstrate that the presence of a biomaterial significantly influences the average velocity of passing leukocytes (*p* < 0.0001; Figure 3a). Especially, in the presence of P(4HB) and PLLA/PCL leukocytes move faster than in the presence of PLLA (P(4HB): 394.0 ± 28.5 µm/s, *p* = 0.042; PLLA/PCL: 496.1 ± 28.8 µm/s, *p* < 0.0001; PLLA: 335.5 ± 26.6 µm/s), while PLLA/P(4HB) reduces leukocyte velocity significantly compared to PLLA (PLLA/P(4HB): 250.7 ± 21.0 µm/s, *p* = 0.007). In addition, the velocity distribution of leukocytes is shown in Figure 3b. Only the copolymers PLLA/P(4HB) and PLLA/PCL do not promote very slow movement of leukocytes and thus tight interactions with the polymeric surface (PLLA/P(4HB): 20.8% < 200 µm/s and 79.2% > 200 µm/s and PLLA/PCL: 6.9% < 200 µm/s and 93.1% > 200 µm/s).

### 2.4. Monocyte Activation

**Figure 4:**
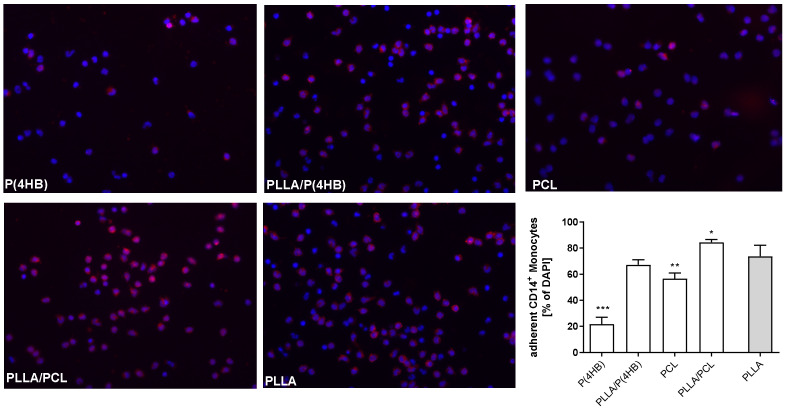
Monocyte adhesion and activation on polymer surfaces. Human peripheral blood mononuclear cells (PBMCs) were isolated from an in-line filter system for leukocyte filtration. Subsequently, PBMCs were exposed to the indicated polymer surfaces for 3 h. After incubation, adherent PBMCs were stained for DAPI (nucleus) and CD14, which is expressed by monocytes. Representative images of 5 independent experiments are shown. Images were quantified using ImageJ software. Bars show mean ± SD of adherent monocytes compared to the PLLA surface with * *p* < 0.05, ** *p* < 0.01 and *** *p* < 0.0001.

Biomaterials face an inflammatory environment upon implantation, which represents a potential obstacle to their success [21]. Monocytes are recruited to the implanted surface during the acute inflammatory phase. Once differentiated to macrophages, they attempt to degrade the biomaterial. Hence, we further investigated monocyte adhesion to the materials under static and flow conditions (Figure 4). Results show that the polymers significantly influence monocyte adhesion (*p* < 0.0001). Especially, P(4HB) (21.7 ± 5.2%; *p* < 0.0001) and PCL (56.7 ± 11.9%; *p* = 0.004) are potentially less adhesive for monocytes under static conditions compared to PLLA (73.8 ± 8.4%). Monocyte adhesion on PLLA/P(4HB) is similar to PLLA (67.2 ± 11.5 %; *p* = 0.455). On a PLLA/PCL surface we counted significantly more monocytes (84.4 ± 10.1%, *p* = 0.041) compared to PLLA.

**Figure 5:**
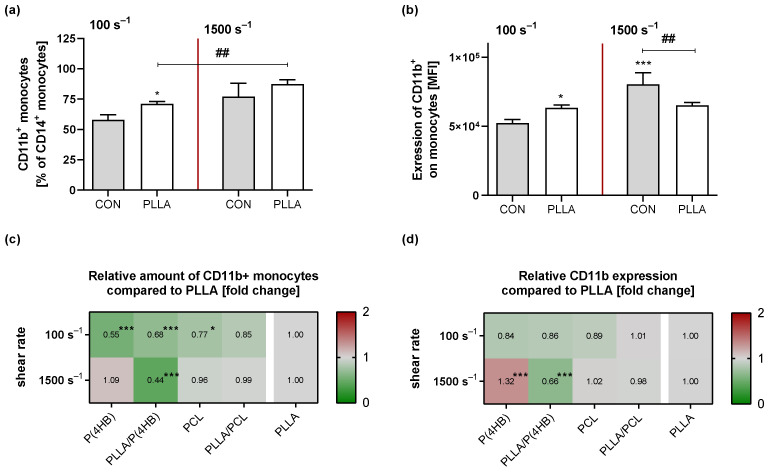
Monocyte activation on polymer surfaces under hemodynamic forces. Human peripheral blood mononuclear cells (PBMCs) were isolated from an in-line filter system for leukocyte filtration and exposed to the indicated polymer surfaces using shear rates of 100 s^−1^ and 1500 s^−1^. Subsequently circulating PBMCs were characterized regarding their CD11b expression by FACS analysis. (**a**) Bars show mean proportion of CD11b+ monocytes ± SD after exposure to the indicated surfaces compared to the control (CON). (**b**) Bars show mean fluorescence intensity (MFI) of CD11b+ monocytes ± SD compared to the control. (**c**/**d**) To visualize the effect of the different polymers, heat maps show the relative mean compared to PLLA. All experiments were repeated 5 times. Significances in regard to the corresponding control are indicated with * *p* < 0.05, and *** *p* < 0.001. Significances between groups are indicated with ## *p* < 0.01.

Further we assessed monocyte activation under static and flow conditions by measuring surface CD11b expression on monocytes via flow cytometry. As described for platelets, we first compared monocyte activation in response to shear forces in a polymer-free experimental setup (Figure S2). Data show an increased number of CD11b+ circulating monocytes due to high shear rates (no shear vs. 1500 s^−1^: 49.6 ± 11.5% vs. 77.2 ± 10.9%; *p* = 0.003) accompanied by an enhanced CD11b expression (no shear vs. 1500 s^−1^: 5.3 ± 0.7 × 10^4^ vs. 8.3 ± 0.9 × 10^4^; *p* < 0.0001). The presence of PLLA influenced the amount of circulating activated monocytes (*p* = 0.002; Figure 5a). Under both flow conditions the percentage of activated monocytes in the presence of PLLA is higher compared to the control (CON vs. PLLA; low flow: 57.9 ± 4.2% vs. 71.2 ± 1.9%, *p* = 0.037 and high flow: 77.2 ± 10.9% vs. 87.4 ± 3.7%, *p* = 0.067). In addition, expression of CD11b on circulating monocytes is influenced by the presence of PLLA (Figure 5b). In response to low shear rates, we observed an increased CD11b expression in presence of PLLA (CON vs. PLLA: 5.2 ± 0.3 × 10^4^ vs. 6.3 ± 0.2 × 10^4^; *p* = 0.024). Interestingly, CD11b expression was significantly reduced under high flow conditions in the presence of PLLA compared to the control (CON vs. PLLA: 8.1 ± 0.8 × 10^4^ vs. 6.5 ± 0.2 × 10^4^, *p* = 0.0003).

Under hemodynamic conditions the amount of CD11b expressing monocytes is influenced as much by the shear rate applied (*p* = 0.001) as by the underlying material (*p* < 0.0001) with a significant interaction for both parameters (*p* < 0.0001; Figure 5c). Especially under low flow, results show a reduced amount of circulating, CD11b expressing monocytes with increasing wettability (P(4HB): 0.55 ± 0.08, *p* < 0.0001; PLLA/P(4HB): 0.68 ± 0.16, *p* = 0.001; PCL: 0.78 ± 0.02, *p* = 0.023; PLLA/PCL: 0.85 ± 0.30, *p* = 0.139) compared to PLLA. Surface expression of CD11b on circulating monocytes is also influenced by the shear rate (*p* = 0.004) as well as by the underlying material (*p* < 0.0001) with a significant interaction for both parameters (*p* < 0.0001; Figure 5d). Under low flow, the expression of CD11b tends to be reduced with increasing wettability (P(4HB): 0.84 ± 0.11, *p* < 0.062; PLLA/P(4HB): 0.86 ± 0.08, *p* = 0.127; PCL: 0.89 ± 0.06, *p* = 0.356; PLLA/PCL: 1.01 ± 0.08, *p* = 0.999) compared to PLLA. After exposure to high shear rates, the presence of P(4HB) leads to a slightly higher amount of CD11b expressing monocytes (1.09 ± 0.01; *p* = 0.501) with significantly increased CD11b surface expression (1.32 ± 0.08; *p* < 0.0001) compared to PLLA. PLLA/P(4HB) shows a significantly reduced amount of CD11b expressing monocytes (0.44 ± 0.02, *p* < 0.0001) when compared to PLLA as well as an decreased CD11b surface expression (0.66 ± 0.02, *p* < 0.0001).

### 2.5. Platelet–Monocyte Aggregation

**Figure 6:**
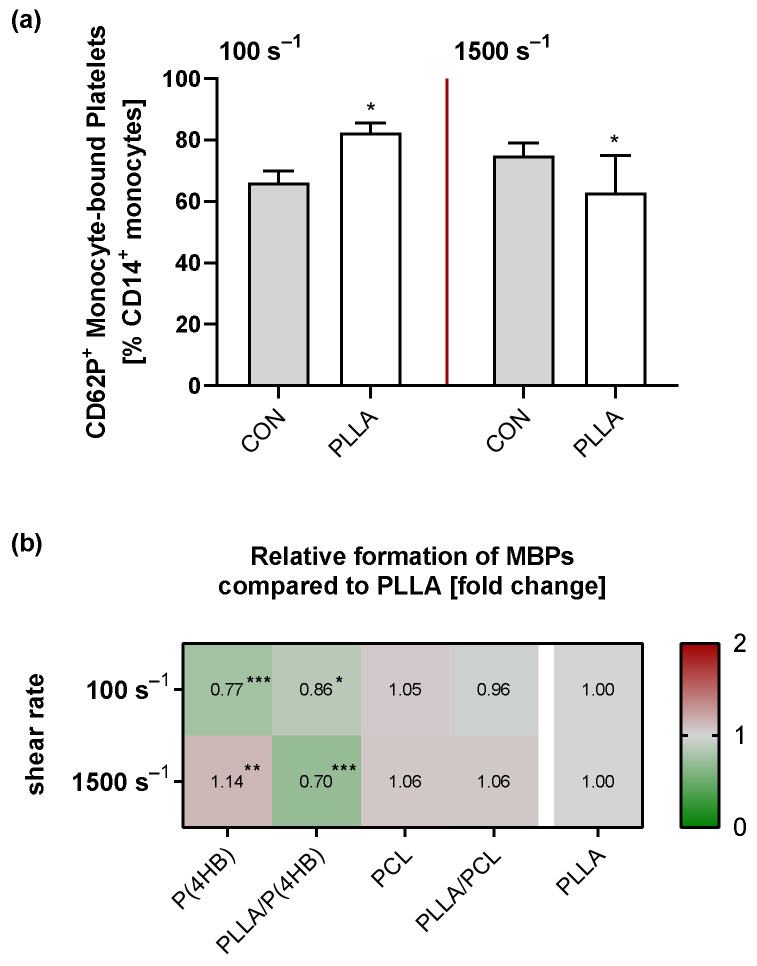
CD62P+ Monocyte-bound platelets (MBPs) on polymer surfaces. Human platelets and leukocytes were isolated separately and resuspended in platelet poor plasma (PPP) at a physiological concentration of 3 × 10^8^ platelets/mL and 5 × 10^6^ leukocytes/mL. Blood cell suspensions were circulated through a parallel plate flow chamber system, containing the different polymers, with shear rates of 100 s^−1^ and 1500 s^−1^. After flow exposure, CD62P+ MBPs were identified within the monocyte population by CD14/CD62P expression. (**a**) Bars show mean proportion of CD14+/CD62P+ MBPs±SD. (**b**) To visualize the effect of the different polymers, the heat map shows the relative mean proportion of CD14+/CD62P+ MBPs compared to PLLA. All experiments were repeated 5 times. Significances with regard to the corresponding control are indicated with * *p* < 0.05, ** *p* < 0.01 and *** *p* < 0.001.

The blood flow pattern within a device and the biomaterial surfaces are well-recognized factors in the development of thrombotic deposition. Importantly, platelets do not only interact with monocytes or ECs at the vessel wall, but platelet–monocyte aggregates (PMAs) are measurable in the circulation [22]. Accordingly, we determined the effect of different flow conditions and biomaterials on circulating monocyte-bound platelets (MBPs) via flow cytometry detecting CD14+/CD62P+ monocytes (Figure S3). The amount of circulating CD62P+ MBPs increases significantly in response to elevated shear rates (no shear: 63.6 ± 6.3%; 100 s^−1^: 71.1 ± 6.1%, *p* = 0.061; 1500 s^−1^: 75.0 ± 4.1%, *p* = 0.006) as well as the expression of surface CD62P (no shear: 3.2 ± 0.3 × 10^4^; 100 s^−1^: 3.9 ± 0.4 × 10^4^, *p* = 0.217; 1500 s^−1^: 4.3 ± 0.9 × 10^4^, *p* = 0.031). Exposure to PLLA further increases the formation of CD62P+ MBPs significantly compared to the control under low flow (CON vs. PLLA: 66.2 ± 3.7% vs. 82.5 ± 3.0%, *p* = 0.046; Figure 6a). Interestingly, we observed a reduced amount of CD62P+ MBPs under high flow conditions in the presence of PLLA compared to the control (CON vs. PLLA: 75.0 ± 4.1% vs. 63.0 ± 12.0%, *p* = 0.036; Figure 6a). 

With regard to the other polymers tested, we found a significant influence of the applied shear rate (*p* = 0.002) as well as the underlying material (*p* < 0.0001) on the amount of circulating CD62P+ MBPs with a significant interaction for both parameters (*p* < 0.0001; Figure 6b). Under low flow, CD62P+ MBP formation decreases with increasing wettability (P(4HB): 0.77 ± 0.02, *p* < 0.0001; PLLA/P(4HB): 0.86 ± 0.05, *p* = 0.020; PCL: 1.05 ± 0.07, *p* = 0.671; PLLA/PCL: 0.96 ± 0.04, *p* = 0.799) compared to PLLA. Under high flow, however, the formation of CD62P+ MBPs is significantly increased in the presence of P(4HB) (1.14 ± 0.05, *p* = 0.008) and reduced in the presence of PLLA/P(4HB) (PLLA/P(4HB): 0.70 ± 0.10, *p* < 0.0001) compared to PLLA.

## 3. Discussion

In general, the deployment of a stent in the vessel wall is associated with varying local side effects. Stents crush the atherosclerotic plaque, cause vascular de-endothelialization and induce mechanical stress, which can initiate inflammatory reactions [23]. However, the impact that biodegradable polymers may have on blood cell activation and leukocyte–platelet interaction under physiological and pathophysiological conditions in the vessel is still not well understood. Blood compatibility studies often focus on local blood cell-material effects and thus miss the dynamics of blood cell activation in the circulation. Therefore, this study aimed to examine the complex processes of blood cell response to biomaterials under different flow conditions. We show that all aspects included in this study are not only influenced by the material employed with the stent, but also by a complex interaction with local hemodynamic forces.

Immediately after stent deployment, proteins from the blood plasma can adhere to the implant surface. This process is strongly dependent on the physical and chemical properties of the biomaterial and triggers a complex series of strongly interrelated events, including platelet and leukocyte activation and adhesion as well as the activation of complement and coagulation [7,24]. Upon activation, platelets bind to immobilized Fg and release intracellular granules containing, for example, CD62P, which further impacts the activation of circulating platelets and inflammation [24]. In addition, the local shear rates of the blood flow direct the aggregation of passing blood cells [25]. In this study, we observed no evidence of activation in circulating platelets at low shear rates, which has also been shown by others [22]. However, exposure to high shear rates led to an increase in platelet aggregation, accompanied by an increased MFI of activation-dependent CD62P. Elevated shear forces have been shown to cause platelet aggregation and activation through a variety of mechanisms [26]. In quiescent platelets, CD62P is located within the inner membranes of α-granules, which are translocated to the outer membrane upon activation. Rahman et al. assessed CD62P expression in response to shear forces, also showing that CD62P surface expression increases in response to elevated shear rates (>1000 s^−1^). In this study, shear rates as low as 400 s^−1^ did not induce P-selectin expression on the platelet surface compared to a non-sheared control [27].

### 3.1. Platelet–Polymer Interactions Are Influenced by Local Hemodynamic Conditions

The presence of a biomaterial may favour platelet aggregation as well as activation via the intrinsic (contact activation pathway) or the extrinsic pathway, both of which lead to the formation of thrombin and fibrin and eventually to thrombus formation [7,28]. In this context, the term thrombogenicity comprises the activation and adherence of platelets to biomaterials. Surface properties such as the availability of certain functional groups, wettability, and topography have profound effects on cell behavior and subsequent cell adhesive events [25,29,30]. Numerous studies show that the lower the water contact angle, the higher the wettability and thus hydrophilicity of a material. Thus, we ranked the polymers regarding their water contact angles according to increasing hydrophobicity: P(4HB) < PLLA/P(4HB) < PCL < PLLA/PCL < PLLA. 

Hydrophilicity is an important feature of biomaterials, as it can alter protein adsorption. The most important proteins that promote platelet adhesion and aggregation on biomaterials include Fg and von-Willebrand-factor (vWF), with Fg being the most abundant in plasma [24,31,32]. Since vWF- dependent interactions in this context become increasingly dominant at very high shear rates (>10,000 s^−1^) [33], we decided on the measurement of Fg in our study. Our data show that the hydrophobic material PLLA enhances the shear-induced aggregation and activation of platelets compared to a polymer-free control regardless of the shear rate applied. This is consistent with Sivaraman et al. who demonstrate that platelet activation increases with increasing surface hydrophobicity, due to adsorbed plasma Fg [12]. In fact, we measured reduced plasma concentrations of Fg after contact with PLLA, suggesting that Fg is immobilized on the PLLA surface. This may account for the observed stronger platelet aggregation and activation. Further, all examined polymers induce less platelet aggregation than PLLA when exposed to low shear rates of 100 s^−1^. This might be related to their more hydrophilic nature compared to PLLA. In addition, the reduced aggregation is accompanied by higher plasma Fg levels in the presence of more hydrophilic polymers, indicating less protein adsorption. In this context, experimental evidence suggests that proteins preferably adhere to hydrophobic surfaces rather than to hydrophilic surfaces due to the energetic cost of displacing water [12,34,35]. However, our results show that local hemodynamic forces also influence protein adsorption and platelet-polymer interactions. Despite their hydrophilic nature, P(4HB) and PLLA/P(4HB) induced platelet aggregation compared to PLLA at higher shear rates and plasma Fg concentrations were comparable to that of PLLA-exposed plasma, suggesting an increased protein adsorption on the polymer surface compared to low flow conditions. On the other hand, PLLA/PCL induces significantly less platelet aggregation regardless of the hemodynamic forces, and although Fg concentration is comparable to PLLA. In this context, some studies report that the conformational state of adsorbed Fg may actually be of more importance for platelet response [12,36]. In this study, we addressed the activation and collagen-induced aggregability of circulating platelets after contact with the polymer surfaces. These platelets might be of particular interest for downstream interactions with the injured vessel wall, where collagen is exposed to the surrounding tissue. However, the examination of the adhered platelets on the surfaces and the determination of adsorbed plasma protein will be valuable in describing the material-induced effects of biomaterials in more detail in further studies.

Of note, platelet P-selectin expression does not differ between the applied shear rates or the underlying material and remains at a high level compared to the polymer-free control, indicating globally enhanced platelet activation in the presence of a biomaterial. Indeed, clinical trials report persistently increased platelet activation in patients’ blood after stent implantation [37,38]. Furthermore, this increased platelet activity positively correlates to the 1-year risk of stent thrombosis and myocardial infarction after stent placement [39]. For stents, anticoagulants are not needed except during placement. Aspirin and Plavix (clopidogrel, antiplatelet aggregation) are used after placement (aspirin forever; Plavix for 1 month or 1 year) to control thrombosis,

### 3.2. Biomaterials Facilitate Leucocyte–Polymer Interactions

Recent studies suggest that the presence of activated leukocytes is also required for activation of the coagulation cascade via an extrinsic pathway [7,15]. In this context, we tracked leukocytes, which were circulated in the presence of different polymer surfaces, to determine the average velocity of leukocyte movement as a measure for possible interaction with the polymer surface. The average leukocyte velocity in the presence of the single polymers P(4HB), PCL and PLLA decreases with increasing hydrophobicity, suggesting that hydrophobic materials allow leukocytes to interact more with the material. This is in contrast to results observed in a model system with well-defined chemistries, showing that hydrophobic surfaces generate more favorable conditions with regard to leukocyte velocity and leukocyte activation [30]. We therefore created a velocity profile of the tracked leukocytes in response to the polymers, showing that leukocytes are somewhat more strongly thwarted after contact with (P4HB) and PLLA and to a lesser extent with PCL. The contact of the leukocytes with an implanted material is, however, only transient, but might have severe downstream effects on cells of the arterial wall, facilitating selectin-mediated interactions with the injured endothelium. Thereby, L-selectin-dependent interactions are the most transient, capturing leukocytes with velocities >100 µm/s, P-selectin-dependent interactions occur typically at velocities in the range of 20–50 µm/s, and E-selectin-dependent interactions show characteristic velocities of <10 µm/s [40]. Of note, the copolymers PLLA/P(4HB) and PLLA/PCL do not promote surface contacts with very slow rolling leukocytes. Taken together, the reduction of the velocity of leukocytes in contact with biomaterials, even if it relates to only few leukocytes, may provide a supportive environment for the downstream formation of selectin-mediated interactions underlying leukocyte capture and rolling on the already injured endothelium after stent deployment. 

Adhered monocytes contribute to thrombosis through membrane-associated pro-coagulant properties, including the upregulation of CD11b or the formation of platelet–leukocyte aggregates [15]. Further, the adherence of monocytes induces an activated phenotype, which strongly influences coagulation due to their pro-inflammatory response [15,41] and data from proteomic studies suggest an altered cytokine secretion, which is dependent on surface chemistry and topography [42,43]. Again, the nature of this process is possibly determined by the adsorbed protein on the biomaterial surface. Tang et al. showed that adsorption and denaturation of fibrinogen allows Mac-1 (CD11b/CD18) positive cells such as monocytes to interact with the material [35]. The more hydrophilic nature of P(4HB), PLLA/P(4HB), and PCL compared to PLLA might therefore contribute to the significantly reduced monocyte adhesion on these biomaterials. 

In addition, we determined surface expression of CD11b which is known to be increased on leukocytes upon activation and is also involved in leucocyte rolling [34] under flow conditions. We observed that shear stress contributes to monocyte activation measured by CD11b expression, which has also been observed in previous studies [22,44]. The presence of PLLA increased the amount of CD11b expressing, circulating monocytes under flow conditions, compared to the polymer-free control. However, under high flow conditions monocytes are activated more in the presence of P(4HB). Data of clinical trials prove the involvement of Mac-1-dependent activation of leukocytes and cytokine secretion in thrombotic complications and restenosis [45,46,47,48]. In vitro studies suggest that the intrinsic pathway alone might not be primarily responsible for platelet activation and thrombus formation [7]. Rather, the activation of adhered monocytes and their pro-coagulant and pro-inflammatory phenotype, which induces the extrinsic activation of platelets, appears to be responsible for material-induced thrombus formation [7,15]. Regarding hemocompatibility, monocyte adhesion and formation of platelet–leukocyte aggregates might thus possess a stronger predictive power than platelet activation alone.

### 3.3. Biomaterials Influence the Formation of CD62P+ Monocyte-Bound Platelets (MBPs)

Activated platelets bind CD62P to monocytes expressing the leukocyte receptor P-Selectin glycoprotein ligand 1 (PSGL-1) [26]. However, recent studies revealed a P-Selectin independent mechanism for the formation of PMAs via glycoprotein Ib [49], which has not been addressed in this study. Therefore, we only refer to CD62P+ MBPs. Activated platelets subsequently induce an inflammatory phenotype in monocytes via P-selectin/PSGL-1 [50] and elevated numbers of such platelet–monocyte conjugates have been suggested to play a crucial role in the pathogenesis of atherosclerosis and inflammatory diseases [29,30]. Recent data suggests that platelet–leukocyte aggregation may facilitate slow leukocyte rolling, adhesion and migration into the vessel wall via Mac-1 [21,23,25]. Since platelet–leukocyte aggregation is determined by blood cell activation [22], high shear rates increase their formation. Additionally, the presence of PLLA induces more circulating CD62P+ MBPs under low flow conditions, which is consistent with our results reported above of enhanced platelet and monocyte activation. However, despite increased platelet activation in response to higher shear forces, the formation of CD62P+ MBP is reduced under high flow conditions. A possible reason might be the reduced CD11b expression on monocytes in the presence of PLLA surfaces. 

**Figure 7:**
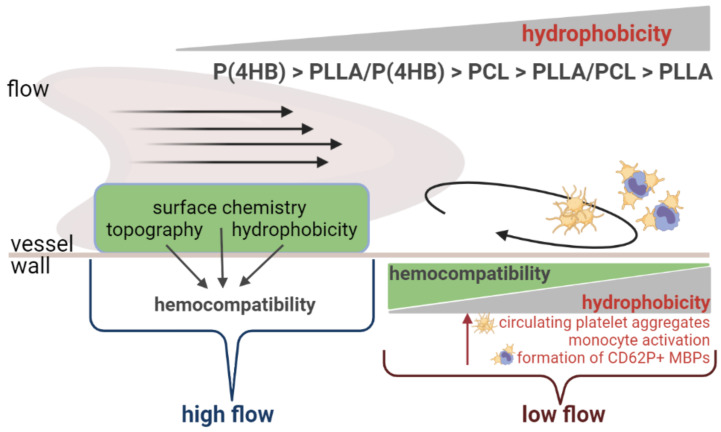
Summary. Material properties such as wettability, surface chemistry or topography are used to predict the hemocompatibility of biomaterials. Our data show that the hydrophobicity of a material is a useful tool for an initial assessment of blood compatibility regarding platelet/monocyte activation and the formation of CD62P+ MBPs under low flow conditions. However, the influence of local hemodynamic forces is often underestimated and does not allow for a rule-of-thumb prediction of the hemocompatibility of a given material.

Overall, formation of CD62P+ MBPs under low flow conditions corresponds to the results for platelet aggregation and protein adsorption and seems to be determined mainly by the wettability of the polymers. We assume that this is due to the reduced activation of platelets and monocytes in the presence of P(4HB) and also to reduced Fg adsorption. Taken together, our data show a complex interaction of hemodynamic forces and the underlying polymers regarding blood cell activation, which is summarized in Figure 7. Thereby, the concept of hydrophilicity/hydrophobicity is suitable for predicting certain properties of blood cell–polymer interactions especially under conditions of low flow, which occur in of recirculation zones and between stent struts.

However, copolymerization of PLLA with P(4HB) and PCL also influenced blood cell–polymer interactions regardless of their wettability. Both copolymers seem to provide the most favorable conditions regarding hemocompatibility. Their surface characteristics might positively influence platelet aggregation and polymer–leukocyte interactions. In fact, a PLLA/P(4HB)-coated stent has already been tested pre-clinically in a porcine model to assess the technical feasibility [51]. Compared to a non-biodegradable metal stent the PLLA/P(4HB)-coated stent performed similar regarding inflammatory response and neointimal hyperplasia. However, with respect to a comparable PLLA-coated stent, inflammatory response and neointimal hyperplasia were significantly improved [51,52,53,54].

## 4. Materials and Methods

### 4.1. Polymer Film Preparation

The following polymer materials were used during film preparation: poly(L-lactide) (PLLA, Resomer® L214, MW = 650,000 g/mol, Boehringer Ingelheim Pharma, Biberach, Germany), poly(4-hydroxybutyrate) (P(4HB), MW = 450,000 g/mol, TephaFLEX®, Tepha Inc., Lexington, MA, USA), poly(ε-caprolactone) (PCL, MW = 80,000 g/mol, Capa™ 6800, Perstorp UK Limited, Warrington, UK). Furthermore, 10 wt% of Triethyl citrate (TEC, Sigma Aldrich, Munich, Germany) was added as plasticizer for the polymeric blend system PLLA/PCL. 

Polymer films were prepared by a pouring process or manual dip-coating as described previously [55,56]. The granules of the pure polymer types ((P(4HB), PCL and PLLA) or the polymeric blends (PLLA/P(4HB) 78/22 wt% and PLLA/PCL 70/20 wt%) were solved in chloroform. The solvent was allowed to evaporate from each sample for over 24 h, and then the samples dried for 1 day at 40 °C in a vacuum drying oven. The dried polymer films were 400 to 450 μm thick. Prior to in vitro testing, all films were sterilized by a common ethylene oxide sterilization process, aerated, and packed sterile. 

### 4.2. Surface Characterization

To assess surface wettability, contact angles were measured by sessile drop method (Contact Angle System, OCA 20, Dataphysics Instruments GmbH, Filderstadt, Germany). Ultra-pure water was used as probe liquid. The resulting contact angles *Θ_W_* were calculated from *n* = 10 samples, which were each determined in duplicates. 

### 4.3. Isolation of Human Blood Cells

Human platelets were obtained as platelet concentrates from the Institute of Immunology and Transfusion Medicine, University Medicine Greifswald, in a polypropylene bag containing acid citrate dextrose A (an anticoagulant solution, 1/6 volume of blood). Platelet-rich plasma (PRP) was obtained by centrifugation at 150× *g* for 15 min. Platelet-poor plasma (PPP) was obtained by centrifugation at 2000× *g* for 15 min. Platelets were counted using Thrombo Count PUR (Servoprax®, Wesel, Germany) and diluted in PPP to a physiological concentration of 3 × 10^8^ cells per mL. 1 mM CaCl_2_ and 0.5 mM MgCl_2_ were added and incubated for 30 min at 37 °C. Freshly prepared PRP was then used for static and shear stress experiments within two hours. 

Human peripheral blood mononuclear cells (PBMCs) were isolated from an in-line filter system for leukocyte filtration (Fresenius Kabi, Bad Homburg, Germany), which was also obtained from the Institute of Immunology and Transfusion Medicine, University Medicine Greifswald. Leukocyte filter were flushed with PBS (w/o Ca^2+^ and Mg^2+^). Subsequently, the cell suspension was pelleted at 500× *g* at 21 °C. Leukocytes were resuspended in 15 mL PBS w/o Ca^2+^ and Mg^2+^) and poured into BioColl-separating solution (Biochrom GmbH, Berlin, Germany) containing Leucosep tubes (Greiner Bio-One GmbH, Kremsmünster, Germany). According to manufacturers’ instructions, samples were centrifuged for 15 min at 800× *g* to obtain an enriched PBMC fraction. PBMCs were harvested, washed trice, and resuspended in RPMI medium containing 10% FBS at a concentration of 5 × 10^6^ cells per mL.

### 4.4. Flow Experiments

Flow experiments were performed at 37 °C using a parallel plate flow chamber (Provitro GmbH, Berlin, Germany) as described previously [55]. Prior to flow experiments, the flow chamber system was flushed with phosphate buffered saline (PBS) for 5 min and with bovine serum albumin (BSA) at a concentration of 4 mg/mL for an additional 30 min. Covalently attached BSA served to passivate the walls of the flow chamber and the tubing by physical adsorption. 

For analysis of platelet–polymer interactions, platelets were isolated as described above and circulated to the flow chamber system at the indicated shear rates for 5 min. 

For analysis of leukocyte-platelet aggregates, platelets and leukocytes were isolated as described above. Subsequently, cells were suspended in PPP at a physiological concentration of 3 × 10^8^ platelets/mL and 5 × 10^6^ leukocytes/mL and exposed to the polymers at the indicated shear rates for 5 min.

### 4.5. Platelet Aggregation

After exposure to shear stress, 2 mL of circulating PRP was collected and collagen-mediated platelet aggregation was measured photometrical using a 4-channel aggregometer (Chrono-Log Model 700, Chrono-Log Corp., Havertown, PA, USA). 500 μL of PRP was analyzed after collagen I (Chrono-Log corp., Havertown, PA, USA) stimulation (5 µg/mL) under constant stirring (1000 rpm) at 37 °C. PRP of the same donor was used as reference and samples were measured in duplicates. Aggregation was quantified by calculating the area under curve (AUC) of aggregation tracings until 6 min after stimulation.

### 4.6. Measurement of Surface P-Selectin and Plasma Fibrinogen (Fg)

Human platelets were isolated and exposed to the different surfaces under laminar shear stress as described above. After perfusion, plasma was collected and assigned to the corresponding analysis method. Surface P-selectin was measured by flow cytometry. Therefore, plasma samples were incubated for 20 min with anti-human CD62P-FITC (eBioscience, San Diego, CA, USA) at room temperature. For Fibrinogen (Fg) measurements after polymer contact, plasma was centrifuged at 1000× *g* for 15 min. Subsequently, Fg enzyme-linked immunosorbent assay (ELISA) (Abcam, Cambridge, UK) was performed according to manufacturer’s instructions. 

### 4.7. Leukocyte Tracking

To assess leukocyte velocity, a suspension of human PBMCs was stained for CellTrace™ Calcein Red-Orange, AM (Life Technologies GmbH, Darmstadt, Germany) and perfused for 5 min with a shear rate of 50 s^−1^ over polymer films, respectively, using a parallel plate flow chamber (Provitro GmbH, Berlin, Germany). PBMCs were detected by immunofluorescence (Eclipse 90i Advanced Automated Research Microscope System (Nikon GmbH, Düsseldorf, Germany), tracked, and the average rolling velocity was determined using NIS-Elements AR Imaging Software (Nikon GmbH, Düsseldorf, Germany).

### 4.8. Monocyte Adhesion

For monocyte adhesion assays, human PBMCs were directly incubated with the polymer surfaces for 90 min under static conditions. After incubation, samples were gently washed, fixed with 4% formaldehyde for 20 min at room temperature, and stained for anti-human CD14-FITC (1:20; Sigma Aldrich, Darmstadt, Germany) and DAPI. For each condition, 10 pictures with 10× magnification were taken in a predefined order. Immunofluorescence was detected by an Eclipse 90i Advanced Automated Research Microscope System (Nikon GmbH, Düsseldorf, Germany) and monocyte counting was done using Image J (NIH, Stapleton, NY, USA). 

### 4.9. Flow Cytometry for the Detection of Monocytes Activation and Platelet–Monocyte Aggregates

Monocyte activation and formation of platelet–monocyte aggregates were assessed under static and flow conditions. Therefore, suspensions of PBMCs and platelets were prepared as described above and directly incubated with the 5 mm Ø control and polymer surfaces for 5 min. Subsequently, the cell suspension was collected and stained with monoclonal antibodies obtained from eBioscience (San Diego, CA, USA) for anti-human CD14-FITC, CD62P-PE and anti-human CD11b-PE-Cy7. Flow cytometry measurements were performed with an Attune® Cytometer (Life Technologies GmbH, Darmstadt, Germany) and analyzed using Attune® Software (Life Technologies GmbH, Darmstadt, Germany).

### 4.10. Data Collection and Statistical Analysis

All experiments were repeated at least five times and representative experiments are shown. If not indicated differently, mean values and standard deviations (SD) were analyzed using Prism 8.3.0 software (GraphPad Software Inc., La Jolla, CA, USA). Main effects of treatments were analyzed using a 1way-ANOVA test. Differences between groups were detected by post hoc analysis using a Dunnett test with correction for multiple testing. Unless otherwise stated, *p* < 0.05 was defined as statistically significant. Main effects of two parameters were analyzed using a 2way-ANOVA with Dunnett post hoc analysis to determine simple column effects.

## 5. Conclusions

Taken together, we show that blood cell activation is strongly influenced by the underlying polymer material and is, in addition to the local hemodynamic conditions, determined by different factors such as wettability, protein adsorption, and surface topography. The combination of altered shear forces and adsorbed plasma proteins on the biomaterial represent an ideal environment for blood cell activation which implies serious consequences for the hemocompatibility of vascular implants. The unmodified materials used in this study face the challenge of considering the activation of coagulation and inflammation at the same time, which makes it difficult to predict hemocompatibility. In this context pre-processed biomaterial with anti-adhesion molecules could be a future approach to prevent biomaterial-induced blood cell adhesion. Considering the complexity of newly developed 3G-DES, we assume that the mechanisms of blood cell-polymer interactions do not allow for a rule-of-thumb prediction of the hemocompatibility of a material, which makes extensive in vitro testing mandatory. 

## Figures and Tables

**Table 1:** Contact angle of water (Θ _W_) on polymers as per sessile drop static measurement. Statistically significant differences to the basic material PLLA were analyzed by 1way-ANOVA.

Polymer	Θ _W_ [°] Mean±Standard Deviation	*p*-Value
P(4HB)	72.1 ± 3.9	<0.001
PLLA/P(4HB)	76.7 ± 2.8	0.001
PCL	78.6 ± 3.2	0.043
PLLA/PCL	79.9 ± 3.4	0.295
PLLA	82.2 ± 1.8	

Θ _W_: contact angle of water; P(4HB): poly(4-hydroxybutyrate); PCL: Poly-ϵ-caprolactone; PLLA: poly-(L-lactide).

## Data Availability

Not applicable.

## Supplementary Materials

The following are available online at https://www.mdpi.com/article/10.3390/ijms22126340/s1.
