# Supplementary material for: The Role of Biodegradable Poly-(L-lactide)-Based Polymers in Blood Cell Activation and Platelet-Monocyte Interaction
Source: Int J Mol Sci. 2021 Jun 13;22(12):6340. doi: 10.3390/ijms22126340 (PMC8231768; doi:10.3390/ijms22126340)
Supplement: Supplementary file 1 [file ijms-22-06340-s001.zip › ijms-1229933-supplementary.pptx]

## Slide 1
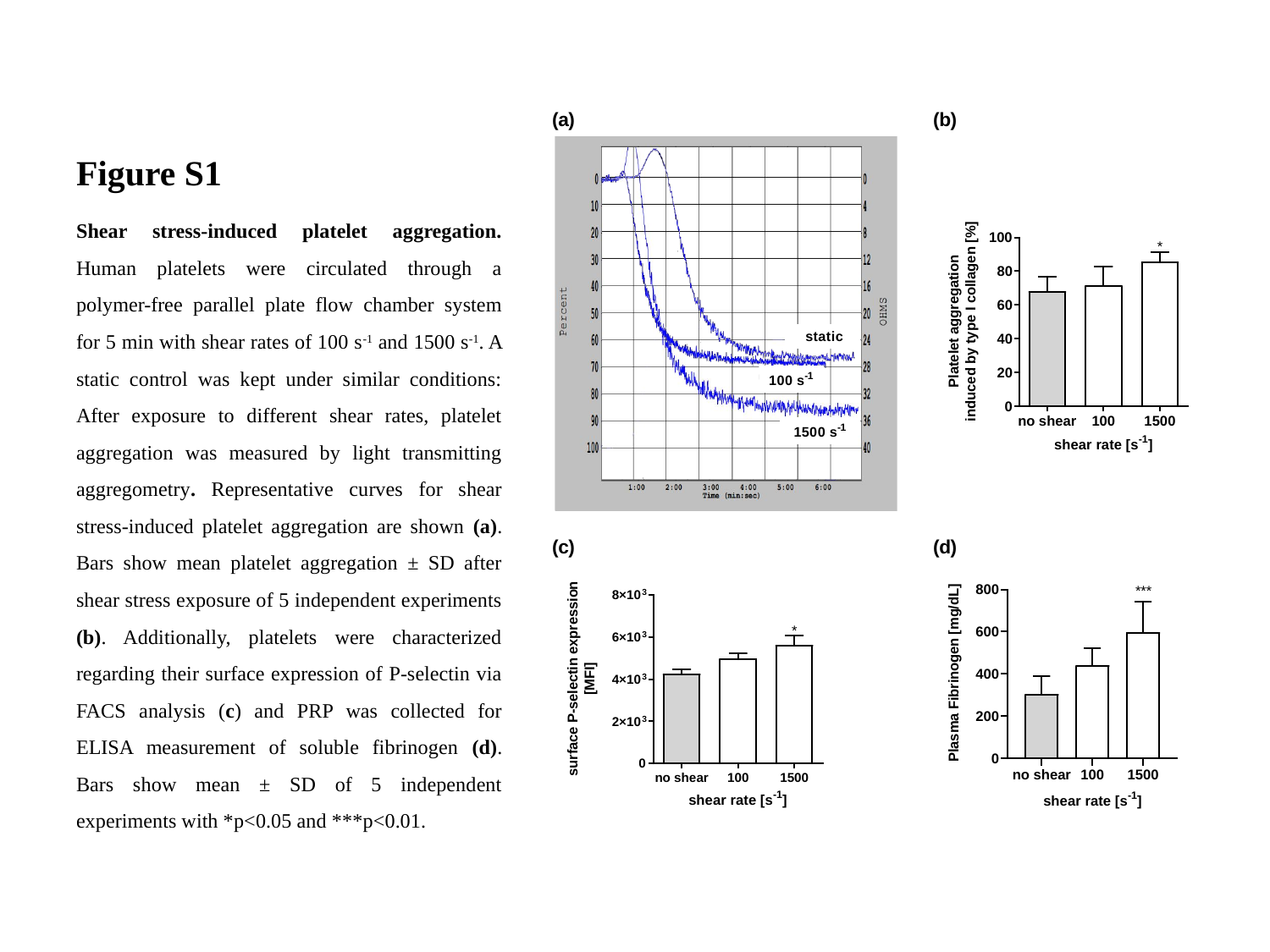

# Figure S1
Shear stress-induced platelet aggregation. Human platelets were circulated through a polymer-free parallel plate flow chamber system for 5 min with shear rates of 100 s-1 and 1500 s-1. A static control was kept under similar conditions: After exposure to different shear rates, platelet aggregation was measured by light transmitting aggregometry. Representative curves for shear stress-induced platelet aggregation are shown (a). Bars show mean platelet aggregation ± SD after shear stress exposure of 5 independent experiments (b). Additionally, platelets were characterized regarding their surface expression of P-selectin via FACS analysis (c) and PRP was collected for ELISA measurement of soluble fibrinogen (d). Bars show mean ± SD of 5 independent experiments with *p<0.05 and ***p<0.01.

## Slide 2
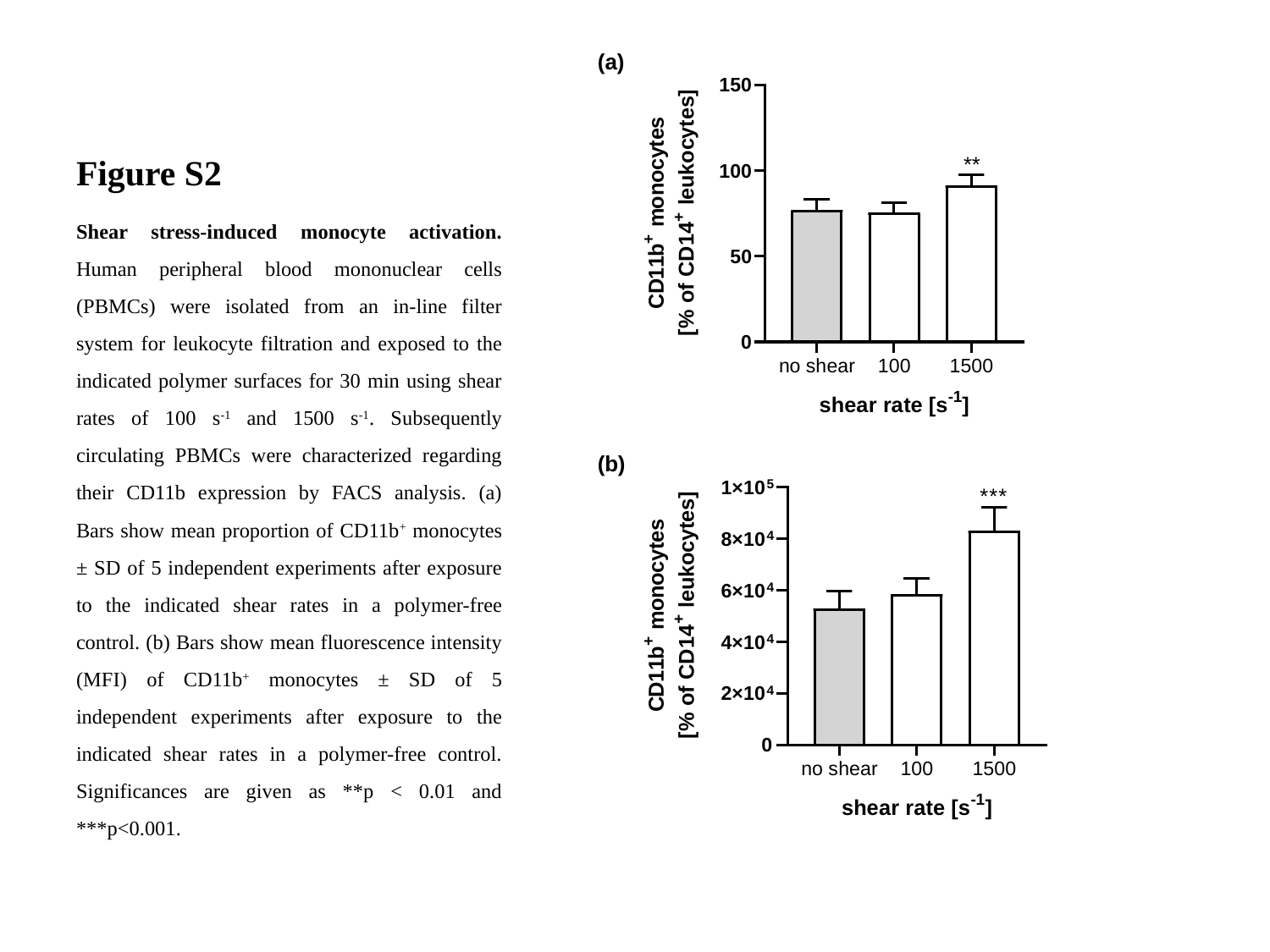

# Figure S2
Shear stress-induced monocyte activation. Human peripheral blood mononuclear cells (PBMCs) were isolated from an in-line filter system for leukocyte filtration and exposed to the indicated polymer surfaces for 30 min using shear rates of 100 s-1 and 1500 s-1. Subsequently circulating PBMCs were characterized regarding their CD11b expression by FACS analysis. (a) Bars show mean proportion of CD11b+ monocytes ± SD of 5 independent experiments after exposure to the indicated shear rates in a polymer-free control. (b) Bars show mean fluorescence intensity (MFI) of CD11b+ monocytes ± SD of 5 independent experiments after exposure to the indicated shear rates in a polymer-free control. Significances are given as **p < 0.01 and ***p<0.001.

## Slide 3
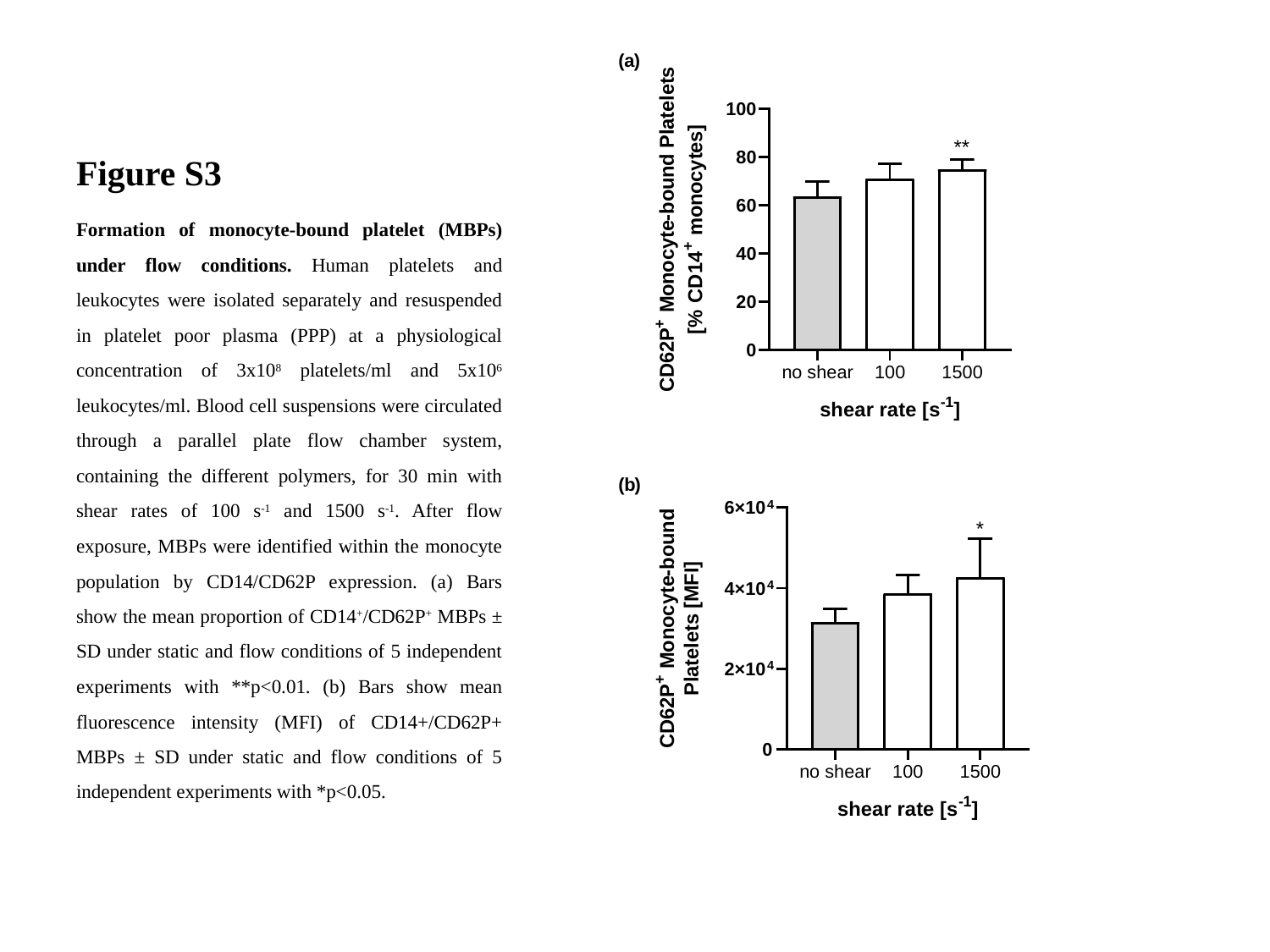

# Figure S3
Formation of monocyte-bound platelet (MBPs) under flow conditions. Human platelets and leukocytes were isolated separately and resuspended in platelet poor plasma (PPP) at a physiological concentration of 3x10^8^ platelets/ml and 5x10^6^ leukocytes/ml. Blood cell suspensions were circulated through a parallel plate flow chamber system, containing the different polymers, for 30 min with shear rates of 100 s-1 and 1500 s-1. After flow exposure, MBPs were identified within the monocyte population by CD14/CD62P expression. (a) Bars show the mean proportion of CD14+/CD62P+ MBPs ± SD under static and flow conditions of 5 independent experiments with **p<0.01. (b) Bars show mean fluorescence intensity (MFI) of CD14+/CD62P+ MBPs ± SD under static and flow conditions of 5 independent experiments with *p<0.05.

## Slide 4
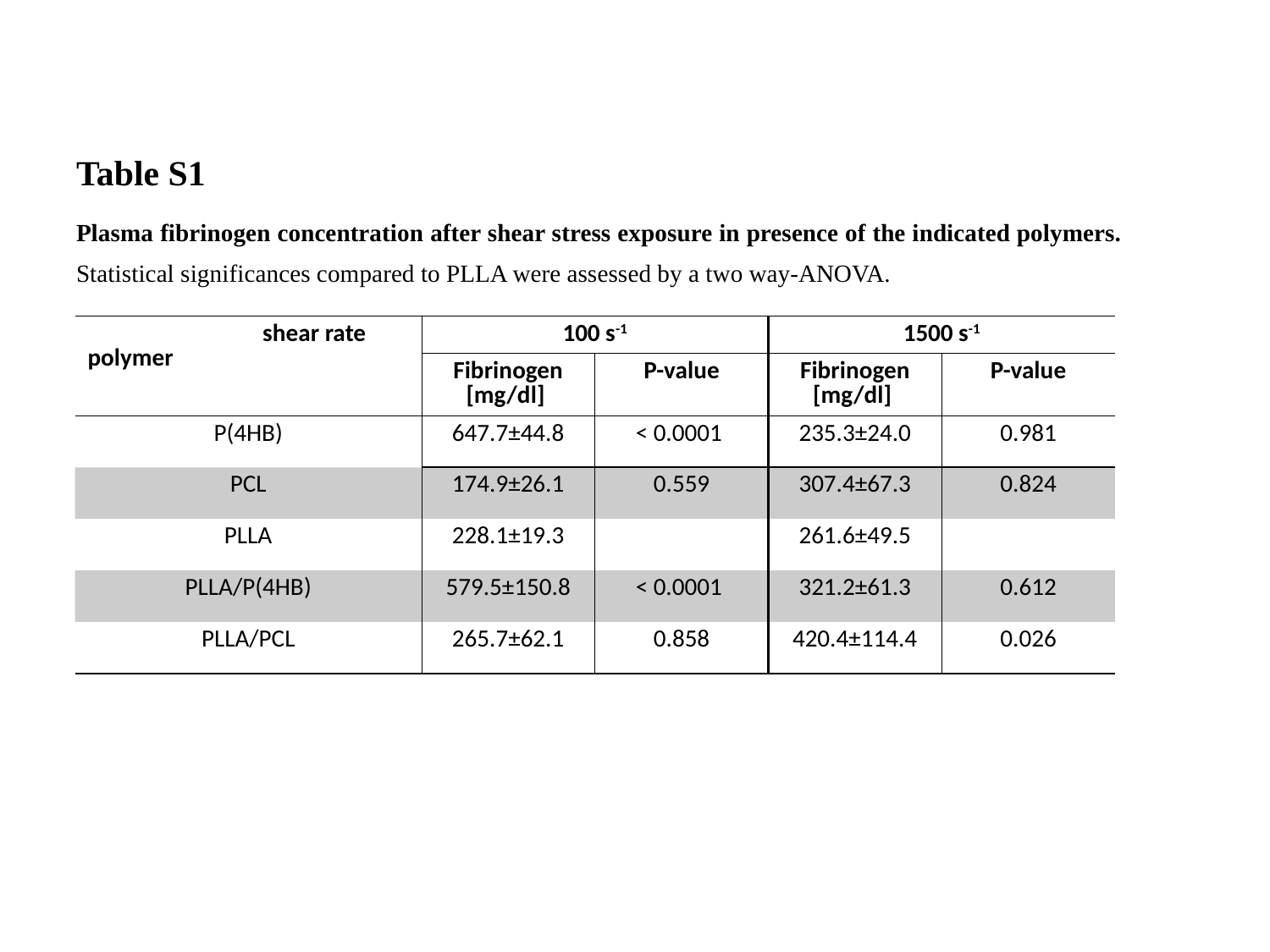

# Table S1
Plasma fibrinogen concentration after shear stress exposure in presence of the indicated polymers. Statistical significances compared to PLLA were assessed by a two way-ANOVA.
| shear rate polymer | 100 s-1 | | 1500 s-1 | |
| --- | --- | --- | --- | --- |
| | Fibrinogen [mg/dl] | P-value | Fibrinogen [mg/dl] | P-value |
| P(4HB) | 647.7±44.8 | < 0.0001 | 235.3±24.0 | 0.981 |
| PCL | 174.9±26.1 | 0.559 | 307.4±67.3 | 0.824 |
| PLLA | 228.1±19.3 | | 261.6±49.5 | |
| PLLA/P(4HB) | 579.5±150.8 | < 0.0001 | 321.2±61.3 | 0.612 |
| PLLA/PCL | 265.7±62.1 | 0.858 | 420.4±114.4 | 0.026 |
